# Early Weight Development of Goats Experimentally Infected with *Mycobacterium avium* subsp. *paratuberculosis*

**DOI:** 10.1371/journal.pone.0084049

**Published:** 2013-12-11

**Authors:** Alyssa N. Malone, Darcy M. Fletcher, Megan B. Vogt, Stephen K. Meyer, Ann M. Hess, Torsten M. Eckstein

**Affiliations:** 1 Department of Microbiology, Immunology, and Pathology, Colorado State University, Fort Collins, Colorado, United States of America; 2 Department of Statistics, Colorado State University, Fort Collins, Colorado, United States of America; Cornell University, United States of America

## Abstract

Johne’s disease is an infectious chronic inflammatory bowel disease in ruminants. The key factor for the management of this disease is an early positive diagnosis. Unfortunately, most diagnostics detect animals with Johne’s disease in the clinical stage with positive serology and/or positive fecal cultures. However, for effective management of the disease within herds, it is important to detect infected animals as early as possible. This might only be possible with the help of parameters not specific for Johne’s disease but that give an early indication for chronic infections such as weight development. Here we report our findings on the development of total body weight and weight gain during the first six months of goats experimentally infected to induce Johne’s disease. Twenty dairy goat kids age 2 to 5 days were included in this study. Goats were divided into two groups: a negative control group and a positive infected group. The weight was obtained weekly throughout the study. Goats of the positive group were infected at the age of seven weeks. We detected significant changes in weight gain and total body weight as early as one week after infection. Differences are significant throughout the six month time period. Weight as a non-specific parameter should be used to monitor infection especially in studies on Johne’s disease using the goat model. Our study suggests that goats with Johne’s disease have a reduced weight gain and reduced weight when compared with healthy goats of the same age.

## Introduction

Johne’s disease, caused by *Mycobacterium avium* subspecies *paratuberculosis* (MAP), is a chronic granulomatous enteritis in domestic and wild ruminants. Johne’s disease poses a significant problem in animal health, which is underscored by its extremely high prevalence in US dairy herds, with 95% and 68.1% prevalence in large dairy herds and dairy operations respectively [1]. Infection usually occurs at birth or during the first months of life through ingestion of contaminated water, milk, or feed. MAP infection can occur in young animals by vertical transmission *in utero* [2]. Johne's disease has a long incubation period, estimated to be 6 months to two years (in some cases even more than five years), and so MAP-infected cattle often go undetected [3,4]. The bacterium replicates in macrophages of the intestinal wall and regional lymph nodes. After an extended incubation period the animal develops a granulomatous inflammation in the distal portion of the small intestine, but also could develop those in the jejunum, at least in small ruminants, that leads to malabsorption, diarrhea, and emaciation. There are four stages of Johne’s disease: (1) silent infection, (2) subclinical infection, (3) clinical infection, and (4) advanced clinical infection [5].

Nielsen and Toft (2009) recently published a meta-analysis on studies focusing on the diagnosis of JD [6]. They classified the tested animals in three groups: (1) affected animals, that shed the pathogen and have clinical symptoms, (2) infectious animals in the subclinical stage, which could also shed bacteria, and (3) infected animals that are considered “latent” or in the silent stage. Infected animals have the least detection rate for current diagnostic tests.

Dairy cattle with clinical Johne’s disease are usually easy to identify; chronic diarrhea, weight loss, and decreased milk production are clear indicators of this disease. The final diagnosis can be provided by additional laboratory testing (e.g. fecal culture, serology), but at this late stage dairy farmers do not need additional testing to help them determine if a cow is sick. Fecal culture is currently the diagnostic gold standard for Johne’s disease. Infected animals can shed a billion bacilli each day, however, some animals exhibit no clinical symptoms (silent and subclinical phase), but still shed the bacteria in feces and milk. Since identification of infected animals via fecal culture is not only expensive but also time consuming, taking weeks to months, new diagnostic approaches are needed [7].

The only effective control measures currently applied by most dairy farmers in the US are culling infected animals and/or instituting good herd management practices. Either approach depends on quickly and accurately identifying MAP-infected animals (preferably during the silent and/or subclinical stage), and separating them from the rest of the herd before they spread the disease through fecal shedding. In recent years several interesting attempts have been made to develop and apply vaccines to efficiently manage this chronic disease in dairy herds. Bastida and Juste provide an excellent overview of the current status on vaccine development and application in the dairy industry [8]. Alonso-Hearn et al. (2012) demonstrated that a heat-killed whole strain vaccine efficiently improves the outcome of the cattle vaccination, although it does not prevent the disease in all animals involved in this particular study [9]. A recent study performed by Cho et al. demonstrated the efficiency of two high-efficiency vaccines in US dairy herds and their effect on the economical performance of the dairy farms involved [10].

The key question for the management of this disease is can infected animals with the greatest potential to develop the disease in the future be identified during the silent and subclinical stage. It seems important to have additional parameters to detect animals infected with the pathogen that are not specific for Johne’s disease but could help to detect animals with a potentially harmful chronic infection. Several parameters could be used including weight and weight development. The scope of this study was to determine the effect of infection with *Mycobacterium avium* subsp. *paratuberculosis* on early weight gain and weight development during the early period of the silent phase of Johne’s disease. Here we report the development of total body weight and weight gain for the first six months after experimental infection of goats and its potential use to detect goats with Johne’s disease during the silent phase of the disease. 

## Materials and Methods

### Animals

Twenty goat kids age two to five days were purchased from a local Johne’s disease-free goat dairy farm section (CCI/Juniper Valley Products; Canon City, CO) and transferred to Colorado State University Campus immediately. The goat kids were housed on Colorado State University Campus (Johne’s disease-free location prior experimental infection) in accordance with Colorado State University animal ethics regulations. Breed distribution of the goats within this study is shown in Table **1**. There were nine Alpine goats with three different sub-breeds (two Alpine-Sundgau, two Alpine-Cou Blanc, five Alpine-Chamoise) and sub-breeds were distributed evenly between groups. This study was approved by the Institutional Animal Care and Use Committee (IACUC) of Colorado State University (#11-3120A). Institutional animal ethics regulations did not permit termination without specific reasons such as severe symptoms (e.g. diarrhea, significant weight loss). 

**Table 1:** Goat breeds/sub-breeds included in this study.

Breed	Number of infected goats	Colorado Registration Numbers	Number of uninfected goats	Colorado Registration Numbers
Alpine	5	6593, 6629, 6583, 6632, 6621	4	6631, 6578, 6617, 6618
Anglo-Nubian	2	6616, 6625, 6682, 6686	1	6622, 6623
Saanan	3	6630	3	6620, 6624
LaMancha	0	6619	1	6619
Toggenburg	0	6627	1	6627

All goats were housed in the same barn until the age of seven weeks and were then split into two groups (infected, uninfected). This barn was cleaned and disinfected before used. No animals with Johne’s disease were housed in this barn before. Each group of ten goat kids was housed in non-adjacent corrals with open barns (fully covered, front wall open, all other walls closed) at the CSU Foothills Campus. All corrals at CSU campus are not attached to other corrals and have space in between the corrals. There were no other animals next to the infected goats and the cows next to the uninfected goats were obtained from Johne’s disease-free herds and were tested for Johne’s disease with negative serological and fecal culture results during this study. The corral of the infected goats housed only Johne’s disease goats prior and during this study. Goats were fully milk fed for 2 months (three times a day). Whole cow milk was purchased from a local store (Walmart, Inc., Fort Collins, CO) in 1-gallon containers. Goats were fed with warm milk in individual feeding bottle with individual nipples used only for the individual goat assigned for. Goat kids were fed individually by hand. Milk feeding was reduced to twice a day for another six weeks and once a day for additional 4 weeks. While weaning, alfalfa hay was introduced to supplement the goats’ nutritional needs. From week 12 after infection, goats were fed with alfalfa hay.

Weights were obtained in pounds (lbs) with a commercially available scale until goats reached 50 pounds. The weight was determined by weighing the person holding the goat minus the weight of the person alone. After this period goat weights were determined with a hanging scale and a calf sling. Weights were obtained on a weekly basis. Weights in pounds were later converted into kg (1 kg = 2.20462 lbs).

### Goat Infection and Inoculum Preparation

*Mycobacterium avium* subspecies *paratuberculosis* (MAP) strain K-10 is a bovine isolate from Nebraska that was provided by V. Kapur (University of Minnesota; now Pennsylvania State University). MAP was grown first on Middlebrook 7H11 agar plates supplemented with 10% OADC (oleic acid, albumin, dextrose, catalase) and mycobactin J (2 µg/ml). Bacteria were then transferred to a liquid culture of Middlebrook 7H9 supplemented with 10% OADC, mycobactin J (2 µg/ml) and 1% glycerol. Cells were washed with PBS (phosphate buffered saline) (pH7.2) and suspended in 20 ml whole cow milk to a final amount of 10^9^ cfu per inoculum. Ten goat kids were inoculated with MAP orally for three consecutive days in compliance with the recommendation of the international committee of Johne’s disease researchers [11]. The bacterial suspension was provided to the goats in a 20-ml syringe capped with the individual nipple of each goat kid. The sterile syringe was not modified for the attachment of the nipple. Goats ingested the whole amount of milk. The infection was performed when the goat kids were 7 weeks old (week 7 or April 10^th^,2012 to April 12^th^,2012). The negative control group received the same amount of normal milk but without the bacterial load.

### Standard Diagnostics of Johne’s disease and clinical observation of infected and uninfected goats

Serum was obtained prior to infection and at various time points after infection (weeks 1, 3, 5, 8, 12, 16, 20, and 24). Serology was performed at the Diagnostic Laboratories at the Veterinary Teaching Hospital at Colorado State University using Parachek (Prionics, Inc; La Vista, NE). Fecal samples were obtained at various points after infection (weeks 1, 3, 5, 8, 12, 16, 20, and 24) and were submitted to the Rocky Mountain Regional Animal Health Laboratory of the Colorado Department of Agricultural Division of Animal Industry for liquid culturing of *Mycobacterium avium* subsp. *paratuberculosis*.

Fecal culture for the identification MAP was performed using standard methods approved by US Department of Agriculture. Briefly, 2 grams of feces is combined with 17.5 ml of sterile water and shaken on a reciprocal shaker for 30 minutes.  After sitting upright for 30 minutes, the top 2.5 ml of each sample is placed in a sterile conical tube and 100 µl of sodium pyruvate is added.  Samples are then incubated at 37 °C for 30 minutes, 2.5 ml of 15% Yeast Extract is added to the sample and incubated for an additional 90 minutes. Then 25mL of a Sterile BHI-HPC Broth and 300µL of 5% Malachite Green solution is added to each sample and incubated 18-24 hours at 37°C.  After incubation, samples are centrifuged at 900 x g for 30 minutes.  In a Biosafety cabinet, supernatant is decanted and to the resulting pellet 1 mL of antibiotic brew (100ug/ml each of Vancomycin and Naladixic Acid and 25 µg/ml of Amphotericin B) is added and samples are incubated overnight (18-24 hours) at 37°C. Inoculate 0.1 ml of prepared sample into each MGIT tube supplemented with Supplement (Bovine Albumin, Casein, Catalase and Oleic Acid), Egg Yolk, Vancomycin, Nalidixic Acid, and Amphotericin B.  Tubes are scanned into the BACTEC MGIT 960 system (Becton Dickinson and Company, Burlington, NC) and incubated for up to 49 days.  Upon signaling positive, the tubes are scanned out and confirmed as positive by both an acid-fast stain and PCR using Johne's VetAlert (Tetracore, Inc., Rockville, MD).

Goats were monitored daily for unspecific clinical signs such as low appetite, diarrhea, inactivity, and/or bloated abdomen.

### Statistical analyses

Statistical analysis was done using SAS 9.3 (Cary, NC). Repeated measures analysis was done using Proc Mixed. The within-subjects factor is week (-6 through 29, excluding weeks 0 and 11) and the between-subjects factor is treatment group (infected or uninfected). A model with arh(1) covariance structure was used, allowing for unequal variances at the different weeks. The main effects of treatment and time as well as the interaction all had p-values < 0.001. Comparisons of mean weights between treatment groups at various weeks were considered. In order to compare weight gains for the treatment groups at different intervals, the weight differences were calculated for each goat at each interval and then a two-sample t-test was used at each interval. A Satterthwaite t-test was used to allow for unequal variances. For both sets of comparisons, a Benjamini-Hochberg adjustment was applied to account for multiple testing [12].

## Results

During the first six months after experimental infection with *Mycobacterium avium* subsp. *paratuberculosis* the standard diagnostic approaches (Parachek, fecal culture) were negative at all time points. Two goats experimentally infected with MAP showed low appetite for the first two days after infection (#6616 and #6629). Intermittent diarrhea was observed with three of the experimentally infected goats (#6621, #6682, #6616) for a few days during the first six months of infection (weeks 20 to 26 after infection). No other clinical signs were observed during the presented time period.

**Figure 1:**
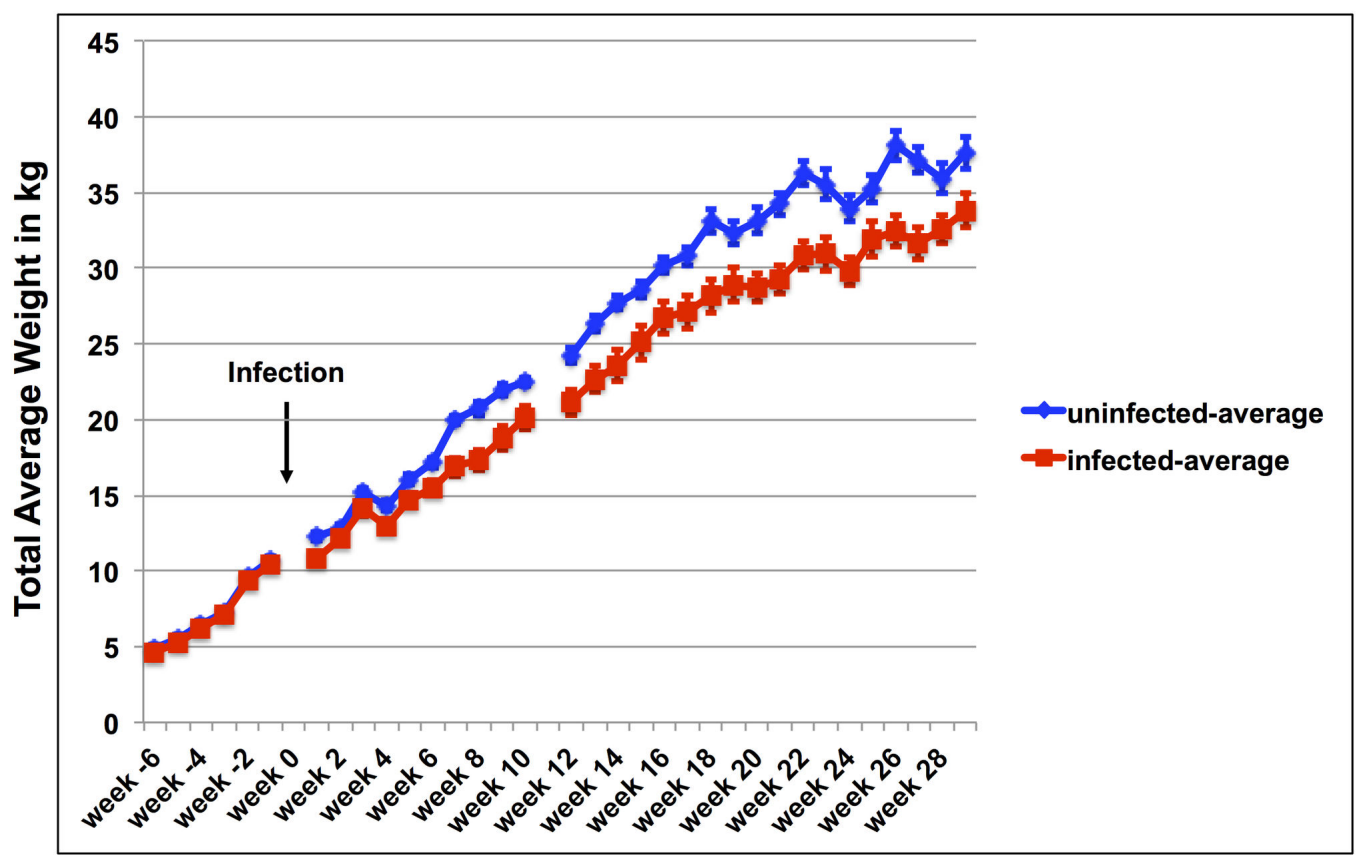
Average weight development of infected and uninfected goats. Development of the average total body weight of goats experimentally infected with *Mycobacterium avium* subsp. *paratuberculosis* (red squares/lines) in comparison with the average total body weight development of uninfected goats (blue squares/lines). The arrow marks the time point of infection. Week numbers reflect the time compared to the time point of infection with negative numbers representing weeks prior to infection and positive numbers representing weeks after infection.

**Figure 2:**
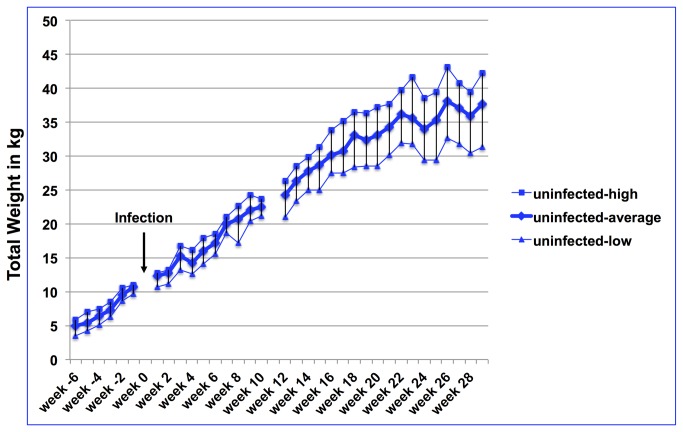
Range of weight development of uninfected goats. Average weight development of uninfected goats together with the highest and lowest weight per measuring point. The arrow marks the time point of infection. Week numbers reflect the time compared to the time point of infection with negative numbers representing weeks prior to infection and positive numbers representing weeks after infection.

**Figure 3:**
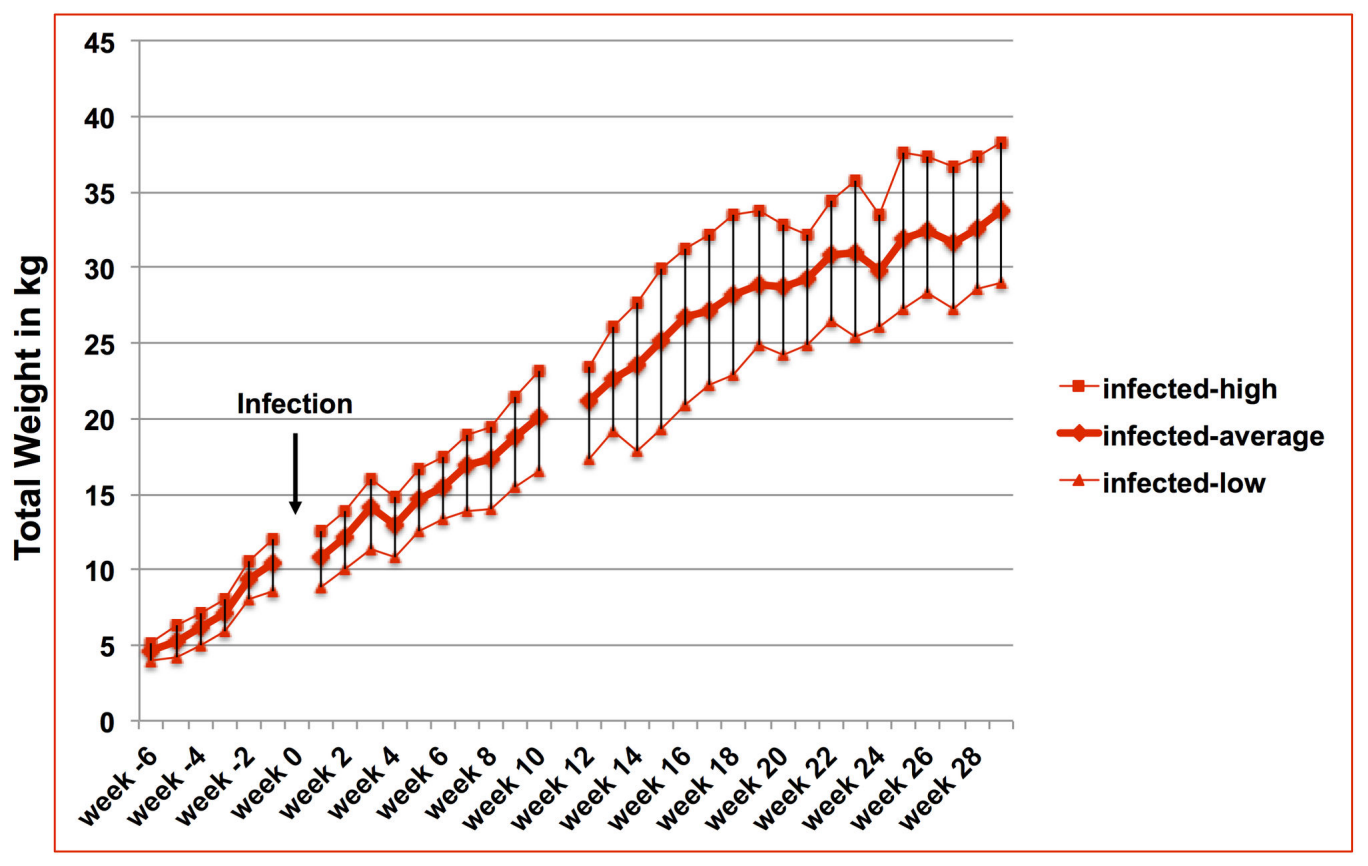
Range of weight development of infected goats. Average weight development of infected goats together with the highest and lowest weight per measuring point. The arrow marks the time point of infection. Week numbers reflect the time compared to the time point of infection with negative numbers representing weeks prior to infection and positive numbers representing weeks after infection.

**Figure 4:**
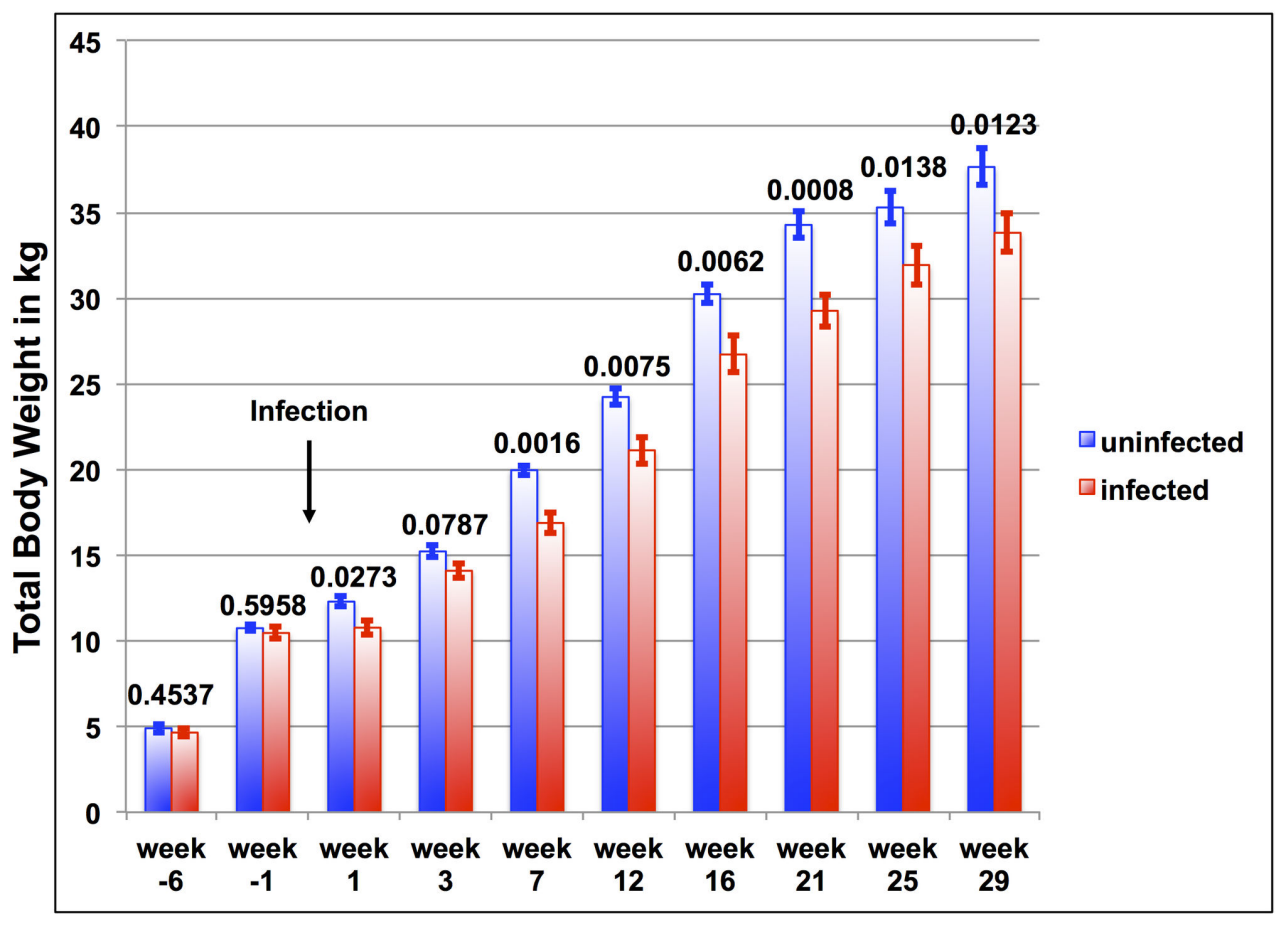
Comparison of the average of total body weight of infected goats (red columns) and uninfected goats (blue columns) of selected weeks. Above each column pair is the p-value corresponding to the comparison. The time point of infection is marked with an arrow. Error bars represent the standard error for each time point.

The actual weight development of each goat (infected and uninfected) was obtained on a weekly basis and the average weight development of infected and uninfected goats is shown in Figure **1**. Weight gain and overall weight development of both groups did not show any differences prior infection. However, after infection, with the exception of a few measuring points, weight differences for most weeks were significant for total body weight. While there was a large variation of weight development in each group (Figure **2** **for uninfected goats;** Figure **3** **for infected goats**) the average weight was significantly different between groups for all weeks except for week 2, 3, and 5 after infection (Figure **1**). Figure **4** demonstrates weight comparisons at selected weeks (two measuring points prior to infection and seven data sets after infection) to illustrate the weight gain reduction early on but almost no weight gain reduction during the later months of infection. Week -6 and -1 do not have statistically significant differences in the total body weight, whereas most measuring points after infection demonstrate statistically significant weight differences.

**Figure 5:**
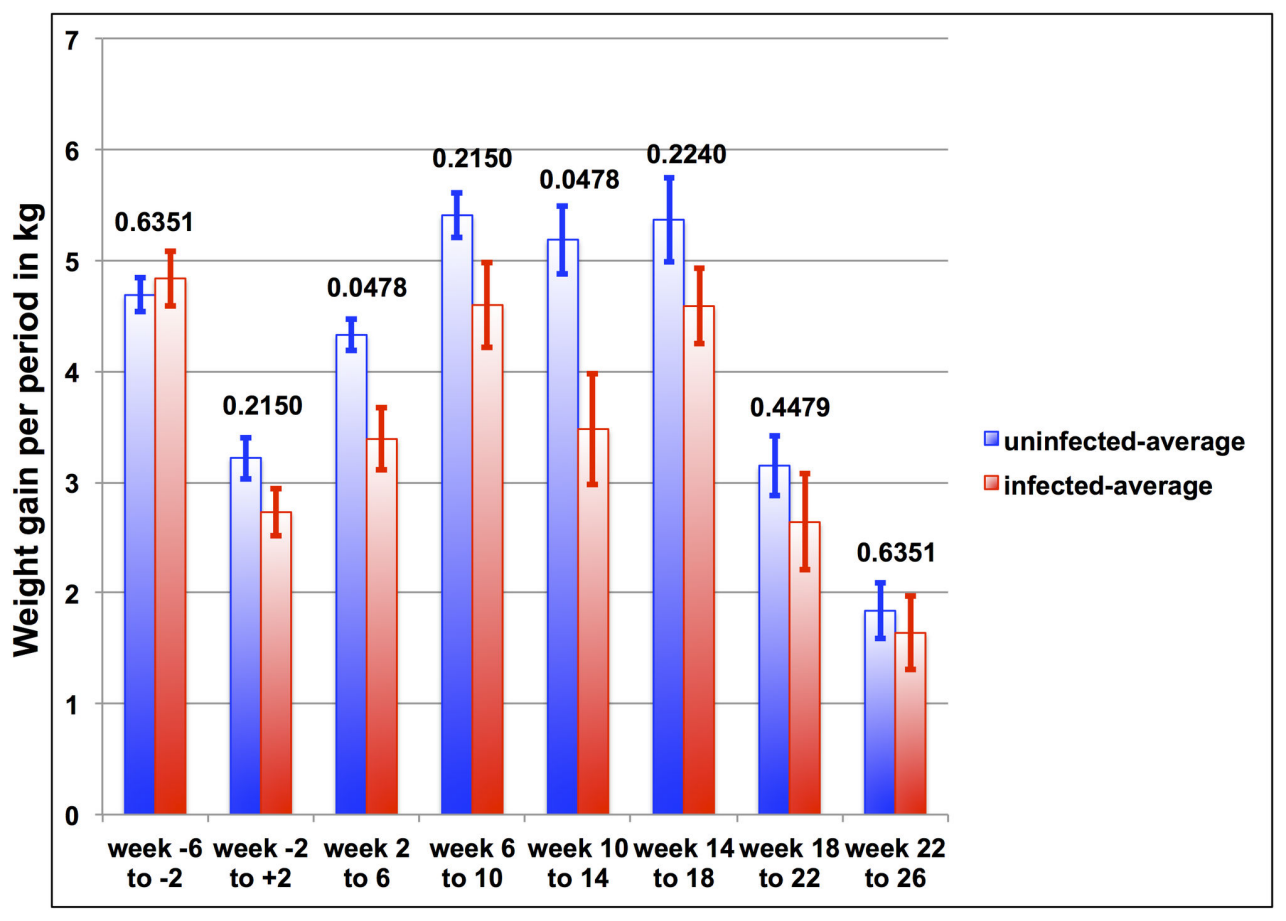
Comparison of average weight gain for four-week intervals of infected goats (red columns) versus uninfected goats (blue columns). There are one interval prior infection, one interval around infection, and six intervals after infection. Above each column pair is the p-value corresponding to the comparison. The infection occurred during the second time interval. Error bars represent the standard error for each time point.

When comparing weight gain per 4-week intervals after infection at every period, the uninfected goats gained more weight than the infected goats (Figure **5**). However, two periods after the infection (week 2 to 6 and week 10 to 14) demonstrate statistical significance with p-values less than 0.05. Further into the infection there is no statistical significance between the weight gain for uninfected and infected goats, although the actual numbers show more weight gain for the uninfected goats than for the infected goats for every period considered here.

## Discussion

No commercially available diagnostic tests exist that allow for the detection of animals infected with *Mycobacterium avium* subsp. *paratuberculosis* during the silent stage; rarely, a few animals can be diagnosed during the subclinical stage through fecal shedding. Thus, it is not a surprise that all commercially available standard diagnostic tests applied in this study were negative throughout the whole six months after infection, similar to results shown by Stewart et al. (2006) [13]. Although this provides us with no evidence that an infection actually occurred, standard infection procedures recommended by an international consortium were applied, which were described as sufficient enough to induce infection in all animals involved [11]. While the standard procedures call for two consecutive days of inoculation, we applied three consecutive inoculations and thus we believe an infection occurred. This hypothesis is supported by the observation of clinical symptoms in two goats during the two days after infection. While intermittent diarrhea could have different causes and in general, diarrhea is rarely seen in goats with clinical Johne’s disease, we attribute the diarrhea to subclinical manifestation of Johne’s disease since none of the uninfected goats developed diarrhea. One has to note that Johne’s disease in goat is more similar to the disease in sheep than in cattle. Although the infection occurs in all domestic ruminants in a similar way and the different phases are seen in all domestic ruminants the clinical symptoms and pathological changes are different in cattle and sheep/goats. While cattle are almost always multi-bacillar and have severe diarrhea, sheep can have either pauci-bacillar forms or multi-bacillar forms and severe diarrhea are rarely seen in sheep [4]. Stewart et al. performed a study with experimentally infected goats in which 3 out of 5 infected goats were identified as infected because of positive fecal culture. Interestingly, for none of them was the clinical disease evident [14]. Surprisingly, they performed a similar study with cattle and none of the experimentally infected cattle had clinical symptoms up to 50 months [15].

 While the scope of this study ended after six months future follow-up with the goats regarding evidence of infection and clinical symptoms might contribute to the findings of this study. Since Institutional animal ethics regulation did not permit termination to confirm infection further observation of the animals is needed to confirm the findings of the study.

Another factor that might have played a role in infection and its association with weight gain and weight development is breed. In our study several different breeds and sub-breeds were used – although equally distributed within the two groups – and breed as well as sub-breeds might have been influential on the weight gain and weight development. However, recommendations by the International Committee of Johne’s disease clearly state that there are no expected differences in the outcome of infections due to breed differences [11]. Furthermore, it is known that different goat breeds have different weight developments and different final weights [16–18]; however, distribution of the different breeds and sub-breeds within the two groups was performed to an almost equal stage to avoid potential differences due to breed and sub-breed differences.

While Johne’s disease could be one of the reasons for reduced weight gain there are other chronic diseases to consider. Several studies were performed to demonstrate the effect of chronic infectious diseases on weight development in ruminants especially in the early stages. Some of them focus on parasite infection such as *Haemonchus contortus* or *Eimeria zuernii*. Rahman and Collins (1991) investigated the effect of host-specific strains (goat-derived versus sheep-derived) of *Haemonchus contortus* on the disease in goats [19]. They observed a strong weight loss for both infected groups, however, a much stronger loss for the group infected with the goat-derived strain. Another study on infection with *Haemonchus contortus* performed by Pralomkarn et al. (1996) focused on the resistance of three genotypes of goats to such infection [20]. While the native goat breed (Thai native) and cross-breed (75% Thai native with 25% Anglo-Nubian) had much less weight gain than the cross-breed with 50% Thai native and 50% Anglo-Nubian, all three breeds gained less weight than their negative control groups. 

Although parasite infections are common and are known to cause changes in weight developments of infected animals our main weight gain differences were seen right after infection and weight gain differences disappeared after two weeks. Since the goats were housed together prior separation into two groups and infection, it is very unlikely that the group of infected goat kids obtained in the new location parasitic infections, although it can’t be ruled out completely.

Weight gain and weight development are non-specific parameters not attributable to any specific disease. Nevertheless, in herds with Johne’s disease, weight gain and weight development could be used to identify individual animals that might develop the disease. Of course, there are different causes that have to be included in any decision, even in our study. However in our study, we did not detect any weight development difference prior to infection; only post infection were weight gain and developmental differences obtained. Our study might come close to the “real world” with outdoor corrals and barns for the goat. The biggest surprise to us was the very early weight gain differences right after infection. These weight gain differences resulted in weight differences that persisted throughout the whole 6-months study. The normal weight gain is about 4 kg per month during the first five to seven months, and 2 kg for next couple of months thereafter [21]. While the goats within the negative control group gained that amount of weight as expected reaching the weight of 37.5 kg, the goats infected with MAP did not gain enough weight to reach the expected weight. Instead, they were on average 3.5 kg lighter than the negative control group. Since the infection with MAP has to occur during the first days after inoculating the goats by invading M cells, epithelial cells, and intestinal macrophages thereafter, it is interesting that this also results in an early reduction of nutrient intake resulting in lower weight gains as expected [22]. Thus, we observed reduced weight gain in the first couple of weeks in the infected group. Later, however, the weight gain differences between the infected and uninfected group were not significant suggesting that the host-pathogen interaction went into the silent phase of Johne’s disease. Kudahl and Nielsen (2009) performed a more comprehensive study on the effect of Johne’s disease on slaughter weight and value of dairy cattle [23]. While previous studies demonstrated an average weight loss of up to 10% body weight and showed that the disease stage has an impact on the weight loss as well with a higher loss in the clinical and advance clinical stage, Kudahl and Nielsen focused as well on the loss of value [23–25]. They demonstrated that, while the weight reduction is clearly seen in their study, there is also a reduction in the muscle mass index for cattle with reduced slaughter weight [23].

More related to our study is the recent study by Elzo et al. (2009) in which they investigated the association of calves and their growth traits with the ELISA scores for Johne’s disease of their mothers [26]. While the effect of cows in the subclinical stage of Johne’s disease on birth weight of their calves was rather small and less significant, calves from ELISA positive cows were significantly lighter at weaning than calves from ELISA negative cows. The reduced weight gain of calves was associated to the disease itself but also to the lower milk production of the infected cows. This latest study demonstrated that the weight development of naturally infected calves is a multi-factorial process and the disease in its silent stage is just one factor. However, if factors such as the lower milk production of infected cows could be eliminated, the pure weight gain of calves could be used to determine animals with the silent Johne’s disease.

Johne’s disease in its early stages appears to have an impact on the overall body weight development of infected ruminants and in particular for this study in goats. It seems to be very helpful to monitor the weight gain per month, especially during the first few months after infection, which naturally occurs right after birth. Both together – weight gain and body weight development – are helpful indicators to identify animals chronically infected including those infected with *Mycobacterium avium* subsp. *paratuberculosis* and will be observed before standard diagnostic tests point towards Johne’s disease. Since our study stands alone on our observation on weight gain and weight development on the goat model for Johne’s disease and the cow model might differ from the goat model at least another study using goats should be performed to confirm our data. Second, another study using calves should be initiated to demonstrate that our findings are equally found in calves infected with MAP.
